# Synthesis, Characterization and Biological Properties of Type I Collagen–Chitosan Mixed Hydrogels: A Review

**DOI:** 10.3390/gels9070518

**Published:** 2023-06-26

**Authors:** Enguerran Devernois, Thibaud Coradin

**Affiliations:** Laboratoire de Chimie de la Matière Condensée de Paris, CNRS, Sorbonne Université, 4 Place Jussieu, 75005 Paris, France; enguerran.devernois@sorbonne-universite.fr

**Keywords:** type I collagen, chitosan, hydrogels

## Abstract

Type I collagen and chitosan are two of the main biological macromolecules used to design scaffolds for tissue engineering. The former has the benefits of being biocompatible and provides biochemical cues for cell adhesion, proliferation and differentiation. However, collagen hydrogels usually exhibit poor mechanical properties and are difficult to functionalize. Chitosan is also often biocompatible, but is much more versatile in terms of structure and chemistry. Although it does have important biological properties, it is not a good substrate for mammalian cells. Combining of these two biomacromolecules is therefore a strategy of choice for the preparation of interesting biomaterials. The aim of this review is to describe the different protocols available to prepare Type I collagen–chitosan hydrogels for the purpose of presenting their physical and chemical properties and highlighting the benefits of mixed hydrogels over single-macromolecule ones. A critical discussion of the literature is provided to point out the poor understanding of chitosan–type I collagen interactions, in particular due to the lack of systematic studies addressing the effect of chitosan characteristics.

## 1. Introduction

Natural macromolecules are particularly attractive starting materials for use to build-up hydrogels for pharmaceutical and biomedical applications [1,2,3]. They are most often biocompatible, i.e., non-toxic, biodegradable and able to trigger specific responses in cells [4]. In the field of tissue repair or regeneration, collagens, and especially type I collagen, are of particular relevance as they constitute the main bio-organic components of many extracellular matrices (ECM) [5,6]. However, many other (macro)molecules play important roles in managing the structural and biological properties of ECM [7]. Thus, the addition of other proteins, glycoproteins or polysaccharides to collagen-based hydrogels offers to enable researchers to prepare more biomimetic materials, which should improve hydrogel therapeutic efficiency [8]. Alternatively, many biopolymers that are not present in human tissues, or are at least present in minor amounts only, can exhibit chemical, structural and/or biological properties that make them useful major constituents parts of biomaterial preparation [9]. In this situation, the addition of collagen may be beneficial, especially in improving or tuning cell–material interactions. Typical examples of such mixed constructs include collagen I–alginate [10] and collagen I–chitosan hydrogels, the latter being the subject of this review.

Chitosan is one of the most popular macromolecules of biological origin in the food, cosmetic, pharmaceutical and medical industries, among others [11]. The reasons for this popularity include the large availability and low cost of chitin, its natural source, and its wide range of biological properties, in particular its antimicrobial efficiency and immunomodulatory effects [12]. Moreover, although neither chitin nor chitosan exists in mammalians, several specific (chitinase) or non-specific (lysozyme) enzymes allow for their biodegradation [13]. Finally, most studies have reported that chitosan is non-toxic in the mg·L^−1^ range. In the field of tissue repair or regeneration, chitosan hydrogels have been evaluated in many areas, but especially for skin [14], bone [15] and cartilage [16]. The uniqueness of chitosan very likely originates from the presence of amine and N-acetyl groups that somehow resemble the carbohydrate chains of proteoglycans. This probably confers chitosan with a higher ability to interact with mammalian cells compared to hydroxylated-only carbohydrates.

Materials associated with collagens and chitosan have therefore been widely described in the literature. However, many of the examined papers refer to membranes or sponges resulting from the drying of mixtures of the two polymers followed by hydration, without evidence of collagen and/or chitosan network formation [17,18,19]. In this context, the aim of this review is to focus on key protocols that have been reported to prepare collagen I–chitosan mixed hydrogels where both macromolecules are gelled in order to provide an overview of their structure and properties and to highlight the benefits of associating these two macromolecules in the field of biomaterials. Apparently contradictory results in the relationship between a material’s composition and its properties call for a better understanding of interactions arising between the two polymers and a more careful consideration of the large diversity of chemical structures that stand behind the term “chitosan”.

## 2. Chitosan and Collagen Hydrogels: A Brief Overview

**Figure 1:**
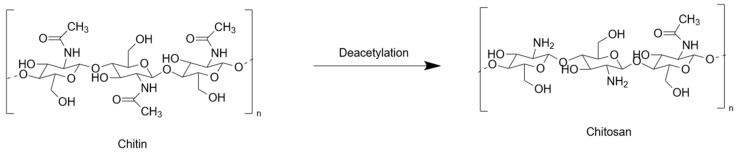
From chitin to chitosan.

Chitosan does not exist as such in living organisms but is a product of the degradation of chitin, the most abundant polysaccharide after cellulose [20]. Chitin is a polymer constituted of 2-(acetylamino)-2-deoxy-D-glucose monomers (often called N-acetylglucosamine) that are linked together by β(1- > 4) glycosidic bonds. Depending on the source organism and extraction procedure, chitin molecular weight usually ranges from ca. 100 kDa to 1000 kDa [21]. The conversion of chitin into chitosan relies on the deacetylation of N-acetyl groups. The extent to which this occurs is usually referred as the degree of deacetylation (DD or DDA), i.e., the percentage of glucosamine monomers (Figure 1). However, some authors rather refer to the degree of remaining N-acetylated groups (DA). Thus, chitin has a DD (or DDA) = 0% and a DA = 100%, but it is often considered that the name ‘chitosan’ applies only to deacetylated chitin with a DDA of ca. 50% or more. When deacetylation is performed by chemical methods, i.e., acidic or alkaline treatments, depolymerization also occurs via hydrolysis, which decreases molecular weight. In contrast, it is possible to independently achieve deacetylation or depolymerization using specific enzymes. Chitosans are usually classified as chitosan oligomers (COS), low MW, medium MW and high MW, although the limits between different categories are highly author- or supplier-dependent. Noticeably, because the direct measurement of chitosan MW is not always straightforward, it is usually calculated from viscosity measurements. However, because chitosan solution viscosity also depends on DDA, the accuracy of such determinations can be low. As a matter of fact, properties of chitosan depend as much on MW as on DDA [22]. For instance, low MW and high DDA increase chitosan water solubility. This can be understood considering that long chains, forming coiled rather than linear structures, and chains exhibiting a low density of free amine groups both show limited hydration and enhanced hydrophobic characters [23]. However, even when these two parameters are optimal, acidification is most often necessary to solubilize chitosan thanks to the full protonation of deacetylated amines. The opposite reaction, i.e., the neutralization of acidic chitosan solutions decreases the charge of the chitosan chain and thus allows for the enhancement of attractive hydrophobic interactions while decreasing repulsive electrostatic interactions, a process that leads to the formation of a physical gel.

**Figure 2:**
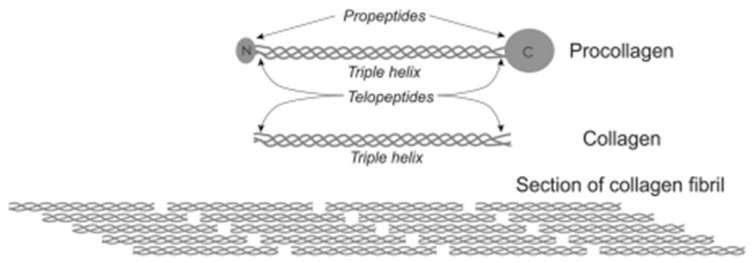
From procollagen to collagen fibrils. Reproduced from [25]—CC B Y 4.0.

Type I collagen is the most abundant protein in most animal tissues [24]. It is a member of the collagen family but, being by far the most widely used in biomaterials, is often simply termed “collagen”. Type I collagen is initially synthesized as procollagen, constituted of three protein chains that form a triple-helix structure with terminal propeptides and telopeptides. The enzymatic cleavage of the propeptides leads to the formation of tropocollagen, the form of type I collagen that can be extracted from tissues (Figure 2) [25]. It has a MW of 300 kDa and an isoelectric point close to pH 8. It is soluble in acidic conditions and undergoes fibrillogenesis upon neutralization [26]. The resulting fibrils further aggregate into fibers to form a hydrogel. Similar to the case of chitosan, the decrease in repulsive electrostatic interactions is partly responsible for gel formation. However, in the case of collagen, hydrophilic rather than hydrophobic intermolecular interactions drive the self-assembly process. The fibrillogenesis process occurs in a similar manner for all type I collagens, independently of their origin. However, differences in amino acid sequences, which are small among mammalians but can be more important in other animals such as fish or sponges, can influence intermolecular interactions and thus the stability of the triple helix [27]. Importantly, telopeptides also play an important role in the self-assembly process. However, they are the main locations of immunogenic moieties. Thus, it is possible to enzymatically cleave telopeptides of tropocollagen, which also makes the triple-helix soluble at a neutral pH environment, but the resulting atelocollagen has limited fibrillogenic capacity [28]. Unfortunately, both tropocollagen and atelocollagen are often referred as ‘collagen’ both by suppliers and authors in the literature.

It is very difficult to accurately compare chitosan and type I collagen hydrogels because there are very few studies that have systematically evaluated the effect of MW and DDA on the properties of chitosan hydrogels. For example, one study showed that storage the modulus *G’* decreases as DDA increases, highlighting that the gel stability results from a balance between hydrophobic and electrostatic interactions [29]. However, *G’* depends on many other parameters such as the initial acidic solution, the ionic strength and the method of gelation for both polymers. In terms of morphology at the microscale, it is relatively easy to identify the presence of well-defined collagen fibrils or fibers, but their width and length can vary greatly with concentration and gelation conditions. In contrast, chitosan hydrogels with a wide variety of microstructures have been reported that are often dependent on the hydrogel drying technique.

In terms of biomedical applications, type I collagen hydrogels are used in almost all areas of tissue repair and tissue engineering due to type I collagen’s natural abundance in living tissues and widespread available commercially [30]. The main limitation of collagen-only hydrogels is that their fast biodegradation is usually improved by chemical cross-linking, which also increases their mechanical stability [31]. In the case of chitosan, hydrogels are mainly used for drug delivery applications thanks to a high positive chitosan charge, good mucoadhesion properties and slow degradation rate [32]. Chitosan antimicrobial and immunomodulatory properties confer it additional benefits in soft tissue repair, especially wound healing [33]. However, it is worth noting that chitosan-only hydrogels are rarely used and that chitosan is most often chemically modified or mixed with another polymer [14].

## 3. Preparation of Collagen–Chitosan Hydrogels

### 3.1. Biopolymer Sources

Type I collagen was present in a majority of cases from animal sources (rat tails, pork skin) and in the form of tropocollagen. Initial solutions were most often prepared at concentrations of 5–10 mg·mL^−1^ in an acetic acid buffer of 0.1 M. Collagen extracted from marine sponges, human-like collagen (HLC), a recombinant protein with a high sequence similarity with collagen but lacking some post-translational modifications such as glycosylation, and collagen hydrolysates were associated with chitosan [34,35,36]. Hydrochloric acid solutions and a 2-morpholinoethanesulfonic acid (MES) buffer were used as alternative media for collagen dissolution [37,38].

In the examined papers, chitosan’s biological origin was most often not specified. Most chitosans were commercial products [39,40,41]. In two cases, chitosan was extracted and purified by the authors [42,43]. When indicated, molecular weight was mainly in the medium range (100–700 kDa) [39,44] and DDA was in the medium-to-high range (75–95%) [45,46]. Concentrations were usually in the 10–30 mg·mL^−1^ (1–3 wt%) range, prepared in an acetic acid buffer or hydrochloric acid. Chemically modified forms of chitosan included methacrylated glycol chitosan, carboxymethyl–chitosan, sulfated chitosan or angiopoietin-1-derived peptide chitosan [35,39,47].

### 3.2. Protocols for Hydrogel Preparation

#### 3.2.1. Physical Gels

**Figure 3:**
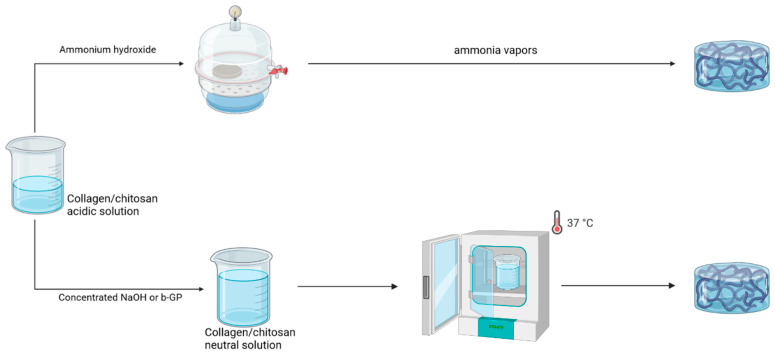
Processes for mixed collagen–chitosan physical gel preparation.

As pointed out above, chitosan and type I collagen have the ability to form hydrogels by neutralizing acidic solutions in common (Figure 3). The use of concentrated NaOH (1 M to 5 M) solutions as neutralizing reagents is the most common protocol for producing either gel and therefore for forming mixed gels. Typically, chitosan and collagen solutions in acetic acid are mixed at room temperature or on ice and NaOH solutions are added until a neutral pH is reached. Gelation is induced by incubation at 37 °C. Noticeably, a neutral buffer can be added before neutralization to allow for both increasing pH, thus limiting the amount of concentrated NaOH solution addition, and the introduction of a cell suspension when necessary [48,49]. Variations in this standard protocol involve neutralization in an ethanolic NaOH solution at −20 °C and induction of gelation by electrodeposition [43]. This method has been mainly applied to mixed gels with major relative contents of collagen and with relatively low polymer concentrations. The main reason for this is that collagen solutions >10 mg·mL^−1^ are highly viscous, meaning that it is difficult to achieve a homogeneous mixing with the NaOH solution before gelation occurs. As a matter of fact, in one study, 15 mg·mL^−1^ collagen solutions were used but were submitted to heating at 50 °C for 1 h. This is very likely to result in partial gelatinization of collagen, as confirmed by the fact that mixed gels formed at 4 °C [50].

A common approach to addressing this issue in the field of collagen hydrogels is to perform neutralization under ammonia vapors [51]. Interestingly, the application of this protocol to chitosan is only quite recent in the literature, the same also being true for mixed gels. In this case, it is possible to use initial solutions with concentrations as high as 20 mg·mL^−1^ for collagen and 30 mg·mL^−1^ for chitosan.

In parallel, protocols that were initially set up for pure chitosan hydrogels have also been applied to mixed hydrogels. The most popular of these is based on the use of β-glycerophosphate (β-GP) [52]. In such protocols, chitosan and collagen solutions in acetic acid are mixed at 4 °C. The addition of a β-GP solution is performed until neutralization and the mixture is incubated at 37 °C to induce gelation. The main benefit of this approach is that it allows for the obtention of thermo-gelling solutions. Moreover, because the addition of β-GP at a low temperature does not induce fast gelation, the protocol can be applied to highly concentrated solutions, as high as 20 mg·mL^−1^ for collagen and 50 mg·mL^−1^ for chitosan [44,53]. This method has been mainly applied to mixed gels with chitosan as the major component. 

#### 3.2.2. Chemical Gels

One of the specificities of chitosan among polysaccharides is that it bears some free amine groups. This opens the possibility of using cross-linking agents that are traditionally used for protein hydrogel preparation. For example, glutaraldehyde was added to acidic mixtures of the two polymers and left to react at RT [54]. Alternatively, the less cytotoxic genipin was also used as a cross-linker for acidic solutions and gelation was achieved at 37 °C [55]. Note that, because the mixture remains acidic, a high polymer concentration can be used. However, in such pH conditions, collagen fibers cannot be formed. The EDC-NHS cross-linking system was also applied to mixed gels [56]. Cross-linkers were added to the polymer mixture before or after neutralization. In some examples, chemically modified chitosans, such as sulfonated-, carboxymethylated- and peptide-grafted chitosans were used. In some cases, cross-linkers consisted of oligomers (polyurethane prepolymers, PUP) or polymers (dialdehyde starch, DAS) [41,57]. In the latter case, the aldehyde functions are expected to interact with both chitosan and collagen. By contrast, in the former case, PUPs are expected to cross-link with the collagen network only. The photopolymerization of methacrylated glycol chitosan–collagen type I neutralized mixtures, performed using riboflavin as a photoinitiator, was also described [39].

Noticeably, several of these reagents were used in alternative procedures where the acidic mixtures are initially dried, most often by freeze-drying, and the following “sponge” was then rehydrated in a solution containing the cross-linking agents.

Finally, it is worth mentioning a double-cross-linking approach that combines physical network formation using β-GP and chemical cross-linking using glyoxal [40]. The two reagents were introduced simultaneously in the acidic polymer mixture at a low temperature and the mixed gels were formed upon incubation at 37 °C. This approach was also used to encapsulate and deliver cells in hydrogels microbeads. The two reagents, β-GP and glyoxal, were mixed together and kept on ice before injection. Hydrogels microbeads were formed using water in oil emulsion using a polydimethylsiloxane (PDMS) bath at 37 °C. Microbeads were then collected and washed [58,59,60].

#### 3.2.3. Enzymatic Cross-Linking

**Figure 4:**
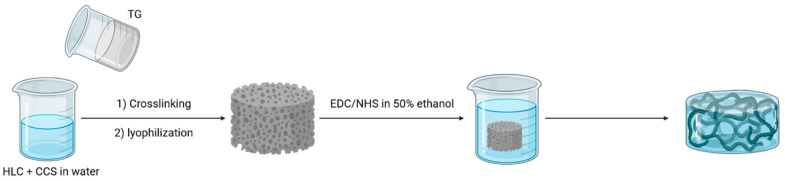
Human-like collagen (HLC)/carboxymethylated chitosane (CCS) using dual crosslinking tranglutaminase (TG) and EDC/NHS.

An original approach was described that aimed at using the HRP (horseradish peroxidase)/H_2_O_2_ enzymatic system to cross-link mixed gels [61]. However, this requires the presence of phenolic groups on the polymer backbone. There are few in collagen (i.e., tyrosine amino acid) and they are absent in chitosan. Thus, the acidic mixture of polymers was first reacted with polyhydroxyphenol (pHP) in the presence of the EDC/sulfoNHS coupling system and, after purification, neutralized with NaOH before the addition of HRP and H_2_O_2_. The transglutaminase enzyme was also studied to design double-cross-linked mixed hydrogels (Figure 4) [35]. Transgluaminase is responsible for collagen cross-linking in vivo by catalyzing the reaction between glutamic acid and lysine. Thus, it cannot link cross-link chitosan chains nor create bonds between chitosan and collagen. Therefore, a two-step process was set up whereby polymer mixtures were first enzymatically cross-linked and, after undergoing freeze-drying, reacted with EDC/NHS [35].

## 4. Characterizations

### 4.1. Gelation Time and Temperature

The influence of polymer mixing on gelation time has scarcely been studied. This is probably because most protocols perform gelation by incubation at 37 °C for a fixed period of time. In the case of chemical cross-linking, it has been reported that chitosan addition to collagen before the latter’s reaction with EDC-NHS increases the gelation time, which suggests that this approach is more efficient at cross-linking collagen than establishing collagen–chitosan links [62]. A similar result was reported for HRP-crosslinked mixed hydrogels [61]. In the case of the β-GP-induced cross-linking, one study reported that adding collagen to chitosan in a 1:2 *w*:*w* ratio decreased gelation time at 37 °C from 12 to 8 min [45]. The gelling temperature was also been studied, showing no or only minor changes when collagen was added to chitosan. These results reflect the fact that collagen alone can form a gel at 37 °C. 

### 4.2. Fourier-Transform Infrared Spectroscopy (FTIR)

Fourier-transform infrared spectroscopy is commonly used to study scaffold composition and identify bonds that make up the system. Studies revealed the presence of bands which are characteristic of chitosan and collagen, including peaks between 3500 cm^−1^ and 3000 cm^−1^, that correspond to the N-H and O-H vibrations of the proteins and polysaccharides. Peaks of amide I (1650 cm^−1^, attributed to C=O vibration stretching), amide II (1590 cm^−1^, attributed to N-H bending vibration) and amide III (1300 cm^−1^, attributed to C-N stretching and N-H in bending) were also found in the spectra of chitosan and collagen. Moreover, a peak between 2200 and 2000 cm^−1^ was noticed. This is characteristic of polysaccharide and does not appear in proteins [50]. In all cases, the spectra of mixed hydrogels displayed bands corresponding to collagen and chitosan. However, some differences appeared after the addition of chitosan or collagen. Using NaOH gelation, a shift in amide I band was noticed that could result from the overlapping of amide bands of collagen and chitosan. Moreover, the addition of chitosan to collagen increased the intensity of the bands of the glycosidic linkages and led to a decrease in the relative intensity of the amine I and amine II bands. These changes were attributed to electrostatic interactions between NH_3_^+^ groups of chitosan and COOH^−^ groups of collagen. In another study, mixed hydrogels formed using NaOH had an intermediate profile between both unitary systems [48]. It is often concluded from such studies that the collagen triple-helix structure is preserved within mixed hydrogels. However, even if FTIR enables scholars to show characteristic patterns of chitosan and collagen, it is difficult to draw unambiguous conclusions about their interactions due to significant band overlapping. Noticeably, this is quite specific to chitosan among other common polysaccharides because it bears protein-like amide groups.

### 4.3. Scanning Electron Microscopy (SEM)

**Figure 5:**
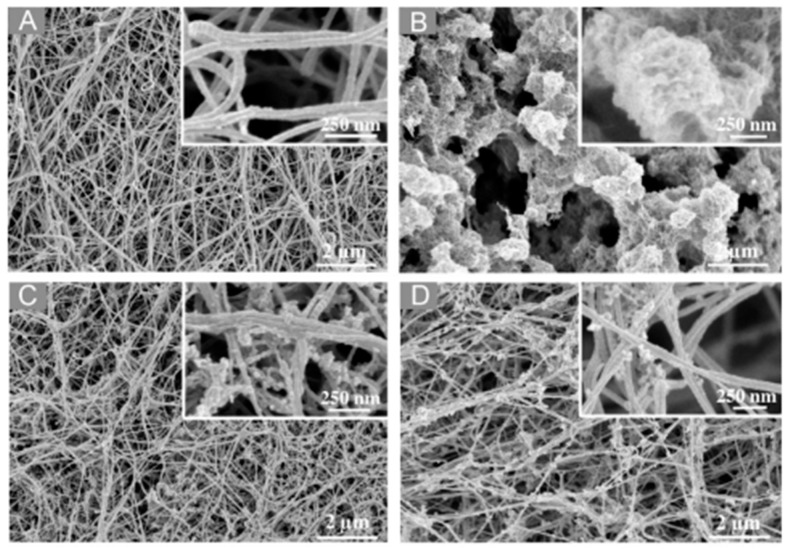
SEM images of NaOH-gelled (**A**) collagen, (**B**) chitosan, (**C**) collagen:chitosan 2:1 *w*:*w* and (**D**) collagen:chitosan 1:1 *w*:*w* hydrogels. Copyright (2011). Reproduced from [42] with permission of the American Chemical Society.

SEM imaging is routinely used to investigate the morphology of hydrogels. However, since these materials are intrinsically composed of mainly water, the sample preparation protocol and, in particular, the drying method have strong impacts on the final morphology of the solid phase. Whereas the well-defined fibrous structure of collagen is somehow preserved in all conditions, chitosan hydrogels after lyophilization can exhibit artefactual sheet-like, honeycomb or large fibrous structures. In this case, which is unfortunately the most common, images of mixed gels mostly show the co-existence of the two networks in a volume ratio proportional to their initial composition. To avoid this issue, it is possible to fix the gel with glutaraldehyde before lyophilization or use critical-point drying. In this case, it is possible to clearly identify mixed networks of collagen fibers and chitosan particles. When collagen is the more abundant polymer, these mixed assemblies form either an apparently interconnected network when chitosan is the major constituent, with collagen fibers seeming to connect chitosan particles together, or adopt a necklace-like structure where chitosan particles are deposited along the collagen fiber surface, [38,46]. Noticeably, these observations were made for different cross-linking methods, suggesting that they do not reflect specific interactions between the two polymers. As a typical example, using critical-point drying, a study of NaOH-gelled hydrogels showed a microstructure made of collagen fibers covered with chitosan particles. Using 2:1 and 1:1 (*w*/*w*) collagen:chitosan ratios, the authors showed increasing fibers size with an increasing collagen ratio (Figure 5) [48]. 

### 4.4. Mechanical Properties

To mimic different situations of use, the mechanical properties of hydrogels are usually studied under conditions of compression or upon stretching. Several studies have suggested that the addition of chitosan to collagen hydrogels improves their compressive modulus scores. For example, the compressive modulus of EDC/NHS cross-linked hydrogels increased in a monotonous way with an increasing amount of chitosan, rising from 175 kPa for a 1:50 chitosan:collagen ratio to 300 kPa for a 1:1 ratio [56]. This trend was confirmed when using the NaOH gelation route and chitosan:collagen ratio of 1:1 and 1:2. This was attributed to the fact that chitosan might hinder the displacement of collagen fibers under the compression stress, thus increasing the modulus [48]. However, in the case of β-GP cross-linking, one study showed that chitosan increased the collagen hydrogel modulus while another reported the opposite trend [45,63]. Interestingly, in the former case, the collagen-only hydrogel had a lower modulus than the chitosan-only gel, while the situation was reversed in the latter case. 

In the case of tensile tests, contradictory results were also obtained. In the case of NaOH-gelled hydrogels, the addition of chitosan decreased the tensile modulus but increased the elongation at the point of breaking. Opposite results were obtained for EDC-NHS cross-linked hydrogels. However, in this case, recombinant collagen was used, that exhibited lower tensile modulus and larger elongation the point of breaking than type I collagen. 

One study used another method that is commonly applied to gelatin gels alone. This technique measures the weight needed by a plunger to depress the surface of the gel of 4 mm without breaking it at a specific temperature. The resulting values are scaled in Bloom and range from 50 to 325. For mixed hydrogels synthetized using the peroxidase-catalyzed crosslinking reaction, higher amounts of collagen (from 1:1 to 3:2 collagen:chitosan ratio) decrease the Bloom strength of the gel [61]. Interestingly, this trend was similar for all investigated HRP concentrations, which confirms that both polymers are involved in the cross-linking reaction. 

### 4.5. Rheological Properties

In the area of hydrogels, rheological studies are mainly performed to study the kinetics of the sol–gel transition and the viscoelastic properties of the material. They are most often carried out under shear conditions by varying a sinusoidal strain at a fixed frequency or frequency at a fixed strain and measuring variations in stress. As a result, storage modulus (*G*′, in Pa) and loss modulus (*G*″, in Pa) are obtained. *G′* characterizes the elastic (i.e., reversible, solid-state-like) response of the gel and *G″* its viscous (i.e., irreversible, liquid-like) response. In principle, there is a linear relationship between storage modulus and Young modulus. However, in the case of hydrogels that consist mainly of water, the relationship between these two properties is not straightforward.

In one study, rheological properties of collagen–chitosan hydrogels crosslinked with genipin were assessed [64]. Chitosan gels had the highest storage modulus and collagen had the smallest one. Intermediate values were obtained for mixed gels and these decreased with increasing collagen:chitosan ratios from 25:75 to 75:25. Noticeably, in another study involving genipin cross-linking where the collagen gels had higher *G′* values than chitosan, the same variation was reported, albeit in the opposite sense (i.e., higher *G′* for higher collagen content) [65]. This would suggest that no interaction exists between the two polymers and that the storage modulus of mixed gels is a combination of those of individual networks.

Interestingly, Sanchez et al. showed that, when collagen–chitosan mixtures were placed at 4 °C before increasing the pH with NaOH, intermediate *G′* values were also obtained for mixed systems [50]. However, when neutralization was performed at 50 °C before cooling, mixed hydrogels showed lower storage moduli than collagen or chitosan alone. In this study, the pure collagen network had a higher *G′* value than the chitosan gel. In the first case, it was proposed that the collagen gel formed first and that then the chitosan network grew in its porosity. Therefore, the storage modulus was mainly imposed by the collagen network. In the second case, it was suggested that the two networks grew simultaneously but that this growth was limited by the presence of the other polymer. These assumptions were supported by studying the thermal stability of the gels at 40 °C. While the gels containing 50:50 collagen:chitosan prepared using the first method behaved similarly to collagen alone, the same gel prepared with the second one was less stable than the two pure hydrogels.

### 4.6. Swelling Properties

Hydrogels have the ability to swell when water penetrates spaces between the polymeric chains. As in vivo environments are highly hydrated, measuring swelling properties can provide some insights on the behaviour of the material after implantation. In addition, they reflect the hydrophilicity of the polymer network as well as its cross-linking density. The most common manner to perform these measurements is to immerge a dried gel in a medium (such as PBS or water) and then to weight the swollen hydrogel at different times. The swelling ratio (in %) is calculated using the equation *SR* = 100 × (*Ws* − *Wd*)/*Wd* with *Ws* signifying swollen weight and *Wd* indicating dry weight. Time intervals for swelling experiments can range from one hour to a few days, with longer times allowing hydrogrels to reach an equilibrated system. Such measurements have been widely reported for mixed collagen–chitosan hydrogels. Using genipin as a cross-linker, increases or decreases in *SR* were reported when collagen was added to chitosan. In one of these studies, the largest SR value was found for a 1:1 collagen:chitosan weight ratio. For HRP-cross-linked systems, an optimal composition was also found, although this was for a 3:2 collagen:chitosan ratio [61]. When β-GP was used, a higher content in collagen led to a higher *SR*. As previously pointed out, these results can strongly depend on the swelling properties of the single-polymer hydrogels. Moreover, experimental time can be as low as 10 minutes, which may not allow hydrogels to reach equilibrium. Finally, *SR* also strongly depends on the cross-linking density and therefore on the gel formation method. The effect of the swelling media was also investigated. In this case, gels (2:1 and 1:1 collagen:chitosan) formed using NaOH were immersed in saline solutions with a high salt concentration (hypertonic solution containing 1.5 M NaCl) or in isotonic solutions (0.15 M NaCl) [66]. *SR* increased with chitosan content under isotonic conditions but decreased with increasing chitosan content under hypertonic conditions. Since the salt concentration of the media had no effect on collagen-only gels, it was proposed that ionic strength modulates the charge of the chitosan chains and therefore the osmotic pressure inside the gels. In contrast, for genipin-cross-linked hydrogels, ionic strength did not significantly influence the *SR* values of mixed gels [67]. Such a difference can be explained by considering that the former are physical gels, whose stability highly depends on physico-chemical conditions, while the latter are chemical gels.

### 4.7. Surface Properties

In addition to the bulk properties of the hydrogels, the characterization of their surface can be of strong interest, especially if they are to be used as 2D cell culture substrates. A first property to consider is the hydrophilic/hydrophobic balance of the surface, which can be evaluated using contact angle measurements. In such experiments, a drop of a given solvent is deposited onto the surface and its spreading is quantified by the angle between the surface and the drop surface curvature at its ends. Although this must be performed with at least one polar, usually water, and one apolar solvent, most studies devoted to hydrogels only report water contact angle measurements. In the case of mixed collagen–chitosan hydrogels, only two studies reported such measurements for genipin-cross-linked materials, showing that chitosan addition to collagen decreases water contact angle, whereas collagen addition to chitosan increases it [68,69]. This finding is consistent with the known hydrophobic character of chitosan, especially for low-DDA chains. 

**Figure 6:**
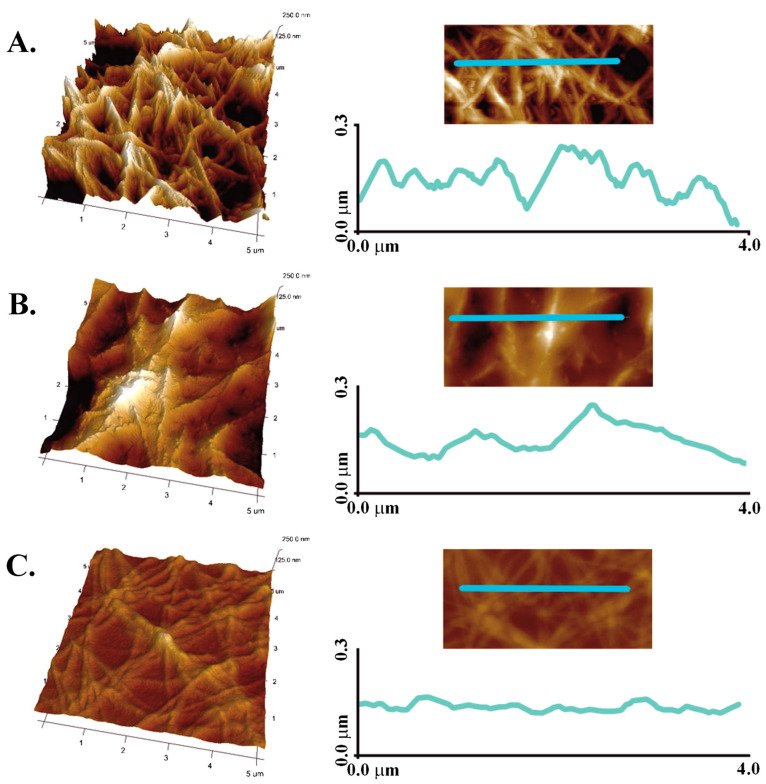
AFM images and rugosity profile of genipin-cross-linked (**A**) collagen, (**B**) collagen:chitosan 1:1 *w*:*w* and (**C**) chitosan hydrogels. Copyright (2018). Reproduced from [58] with permission from Elsevier.

Other important features of hydrogel surface include its topology and local mechanical behavior, both of which can be studied using atomic force microscopy. In one study, collagen, chitosan and 1:1 mixed hydrogels cross-linked with genipin were imaged by AFM (Figure 6) [64]. A pure collagen surface consisted of collagen fibers, with a roughness of ca. 30 nm, whereas pure chitosan showed a particulate structure with a roughness of ca. 25 nm. Interestingly, the mixed gel exhibited a significantly smoother surface (*ca*. 15 nm), suggesting that the collagen fibers were embedded within the chitosan network. Nanoindentation experiments were also performed on mixed hydrogels with 8:2 and 7:3 collagen:chitosan ratios cross-linked with EDC-NHS and they showed that the latter exhibited an elastic modulus 5 times larger than the former [70].

## 5. Biological Properties

In a majority of the studies, one of the polymers is used as an additive to a hydrogel made from the other in order to improve some of its biological properties. In the case of collagen-based materials, chitosan is mostly used as an equivalent of glycosaminoglycans (GAGs). As such, its addition is expected to enhance the binding of key biomolecules, such as growth factors, to the hydrogel as well as to act as a co-receptor in cell signaling processes. In addition, GAGs play an important role in the mechanical behavior of the ECM so that chitosan addition to collagen should provide a more biomimetic environment to the cells. Accordingly, in the case of chitosan-based hydrogels, type I collagen is used to introduce specific biochemical motifs in the matrix that can favor cell adhesion, differentiation and proliferation and tune the mechanical and chemical stability of the hydrogels, including the biodegradation rate.

**Figure 7:**
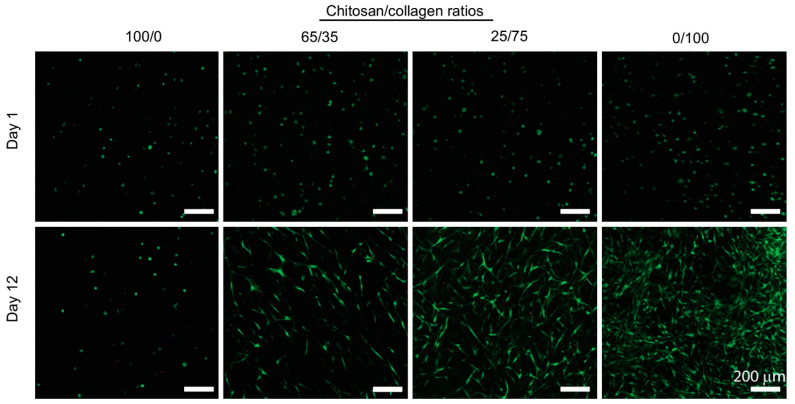
Spreading and proliferation of hBMSC encapsulated in collagen:chitosan hydrogels at various *w*:*w* ratios. Copyright (2010). Reproduced from [66] with permission from Elsevier.

These two approaches are particularly well-illustrated in the field of bone regeneration. Following the first strategy, mixed hydrogels were prepared by NaOH neutralization at a fixed collagen concentration and with final collagen:chitosan *w*:*w* ratios of 1:0, 2:1 and 1:1 [48]. They were used as hosts for MC3T3-E1 murine calvarial osteoblasts that exhibited a higher proliferation in the long term in the presence of chitosan. This result was correlated with a decrease in cell-induced contraction of the hydrogel upon chitosan addition. Additionally, chitosan promoted alkaline phosphatase (ALP) activity, mineral phosphate deposition, and therefore higher osteoblastic activity. However, it was noticed that, in addition to hydroxyapatite, octacalcium phosphate (OCP) was also formed, something that had previously been reported for chitosan alone. Altogether, the beneficial effect of chitosan appears to be mainly related to the structural and mechanical modifications of the hydrogels. In parallel, when chitosan hydrogels were modified by collagen addition in small amounts (collagen:chitosan ratio ca. 1:10), it was commonly observed that mesenchymal stem cells (MSCs) exhibited a better spreading and, in some cases, a higher viability [46]. Most interestingly, the presence of collagen increased ALP activity and mineral deposition in vitro and allowed for ectopic bone formation in vivo. In a series of papers, a wide range of ratios was studied but, in this case, these were prepared by varying the concentration of each polymer [40,45,58]. Therefore, it is difficult to distinguish between the influence of each polymer and that of the mixture. With this limitation, it was found that bone marrow-derived stem cell (BMSC) proliferation was improved compared to chitosan alone for a β-GP-induced gel with a 35:65 collagen:chitosan ratio, but that it did not significantly increase for higher collagen content (Figure 7) [45]. Interestingly, the expression of bone-related proteins such as osterix (OSX) and bone sialoprotein (BSP), as well as ALP activity and calcium deposition, were higher at this specific ratio compared to pure collagen. It was noticed that such composition corresponds to an intermediate gel contraction between chitosan- and collagen-only hydrogels.

**Figure 8:**
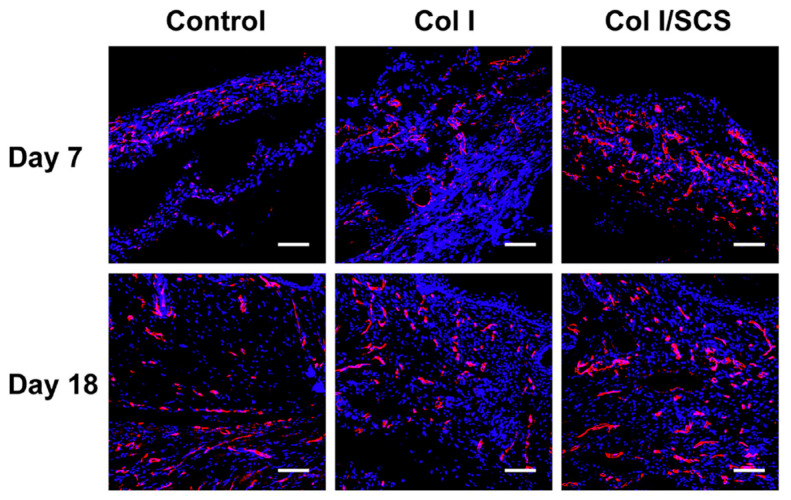
In vivo wound healing in diabetic mice after 7 and 18 days. Fluorescence images after immunostaining with CD 31, in red, and DAPI (in blue) of collagen and collagen:chitosan 1:1 *w*:*w*. Control is a commercial (Tegaderm) dressing. Copyright (2020). Reproduced from [68] with permission from Elsevier.

In the case of soft tissue repair, the benefits of adding chitosan to collagen hydrogels were highlighted for growing chondrocytes, myoblasts and fibroblasts in vitro [63,68]. The introduction of sulfonated chitosan also favored skin wound healing in vivo, which was attributed to enhanced vascularization (Figure 8) [47]. This enhancement was related to the regulation of inflammation through the promotion of interleukine-4 (IL-4) and tumor necrosis factor alpha (TNFα) production. The positive effect of chitosan addition to collagen on angiogenesis was also observed after subcutaneous implantation by promoting endothelial cell differentiation, especially for a 1:1 composition [71]. Again, the ability of chitosan to act as a GAG-like molecule and specifically interact with growth factors was suggested to explain these results. In parallel, the positive effect of collagen addition to chitosan hydrogels on the viability of fibroblastic lines (L929, 3T3) [42,72] and adipose-derived stem cells (ADSCs) [73] was reported, while it did not influence the viability of human skin fibroblasts (HSF) and mouse embryogenic fibroblasts (MEF) [55]. Such a discrepancy may indeed reflect intrinsic features of each cell line, but it also highlights the various concentrations and gelation/cross-linking methods used in these works. Animal studies are scarce but, in one study, injectable chitosan/β-GP gels with a collagen:chitosan ratio of 20:3 and 2:1 ratio were used to decrease post-operational bleeding and improve healing after endoscopic submucosal dissection [67]. Preliminary in vitro evaluations demonstrated that the presence of collagen had a favorable impact on coagulation. Collagen did not improve the viability of seeded-ulcer-healing-related cell lines but increased the production of the epidermal growth factor (EGF) in one of these cell populations. The presence of collagen also favored early inflammation after subcutaneous implantation. However, after 10 days, all hydrogels were fully degraded.

The elaboration of mixed hydrogels was also performed to tune the release of active molecules. For instance, hydrogels with collagen: chitosan ratios of 1:1, 2:1 and 1:10 were used for the delivery of boric acid, thymosin b-4 and fucoidan, respectively, to improve angiogenesis and tissue repair [43,49,74]. Unfortunately, in these studies, variations were made in drug dose and not in hydrogel composition so that it is not possible to draw any conclusion on the benefits of preparing mixed systems. However, in one study, chitosan, collagen and mixed (collagen:chitosan 1:1 and 1:2) gels formed using β-GP were compared for the release of quercetin as an antioxidant agent to periodontal ligament stem cells (PDLSCs) [63]. It was found that the use of chitosan alone led to a very slow release, probably due to favorable electrostatic interactions between the positively charged polymer chains and the negatively charged quercetin, while the release rate was very high from the collagen hydrogel. In parallel, it was found that the viability of PDLSCs decreased with an increasing amount of collagen in unloaded gels. On this basis, the authors selected 1:2 as the best composition based on a compromise between release rate and cytotoxicity.

## 6. Critical Assessment and Perspectives

Altogether, these works validate the hypothesis that mixed systems can be better hosts for a variety of cells and for tissue repair compared to single-polymer hydrogels. However, while the main hypothesis of these approaches is that the two polymers act in synergy from a biological perspective, several studies suggest that there is in fact an optimal composition. This is because many other properties of hydrogels, including mechanical properties, porosity, swelling and biodegradation rate, are also influenced by the collagen:chitosan ratio. Our survey highlights that available data related to such properties often appear contradictory. Most simply, this because the concentrations in each polymer can be very different from one study to another and obviously also because they strongly depend on the gelation/cross-linking method. Last but not least, it was earlier pointed out that both chemical and biological properties of chitosan vary to a large extent with its DDA and MW. With a very few exceptions, the influence of these parameters is not studied or even mentioned in the literature examined here. In fact, in some cases, MW and/or DDA are not provided in the experimental section of the article. Accordingly, although some authors did try to compare several gelation/cross-linking methods [51], there is not enough data available to draw any useful conclusion. As a general trend, physical gels are preferred for 3D cell immobilization as they avoid potentially toxic cross-linkers. More (bio)-chemically stable gels are obtained after chemical cross-linking and enzymatic cross-linking can offer a good compromise between these two approaches. However, how encapsulated cell viability and gel stability are impacted by MW and DDA is also unknown. Other aspects that were not addressed in the reviewed literature are the molecular interactions within mixed networks in physical gels and the extent of chemical cross-linking between the two polymers, both of which will highly depend on the chitosan chain characteristics. Facing these limitations, there is a strong need for more systematic studies that focus on the effect of MW and DDA on the properties of gels prepared with different methods to improve a given property, whether it is structural (i.e., porosity), physical (i.e., mechanical properties), chemical (i.e., degradation rate) or biological (i.e., cell adhesion), for a given application. 

It is also important to emphasize again that this review was limited to the available literature devoted to mixed hydrogel preparation and characterization. As pointed out earlier, mixed membranes or sponges were also widely described because these are the most suitable forms for specific applications. Since their preparation involves a drying step and does not necessarily rely on gel formation, their structural, chemical and mechanical properties are expected to significantly differ from highly hydrated gels. Thus, there would be a strong interest in establishing comparisons between materials obtained by different processes.

Finally, and even more importantly from our point of view, there is in fact very little knowledge about the chemical interactions that can exist between chitosan and type I collagen in solution. In fact, since the pioneering works of Taraval and Domard, these interactions have been sparingly studied, and where they have it has only been in a narrow range of conditions (chitosan type, pH, concentration, …) [75,76,77].

Hence, there is still no global understanding of how these two polymers interact and how it impacts their gelling properties and interactions with cross-linking agents. Such an understanding would certainly be the key to better anticipating the properties of mixed hydrogels and further improving existing protocols, especially for application in the most recent processing techniques such as electrospinning and 3D printing [78,79].

## Data Availability

Not applicable.
